# Type 2 Myocardial Infarction: A Geriatric Population-based Model of Pathogenesis

**DOI:** 10.14336/AD.2019.0405

**Published:** 2020-02-01

**Authors:** Alain Putot, Melanie Jeanmichel, Frederic Chague, Patrick Manckoundia, Yves Cottin, Marianne Zeller

**Affiliations:** ^1^Geriatric Department, University Hospital of Dijon Bourgogne, France.; ^2^Cardiology Department, University Hospital of Dijon Bourgogne, France.; ^3^INSERM U1093 Cognition Action Plasticite, Universite de Bourgogne Franche Comte, France.; ^4^Physiopathologie et Epidémiologie Cerebro-Cardiovasculaires (PEC2), Universite de Bourgogne Franche Comte, France.

**Keywords:** type 2 myocardial infarction, anemia, respiratory tract infection, aortic stenosis, tachyarrhythmia pathophysiology

## Abstract

Distinction between type 2 myocardial infarction (T2MI), defined as an imbalance between oxygen supply and demand without atherothrombosis, and type 1 myocardial infarction (T1MI), due to plaque disruption, is often a clinical challenge in frail elderly patients. We aimed to identify the characteristics and underlying causes of T2MI using a comprehensive geriatric approach. From a multicentre population-based prospective study in coronary care units, we adjudicated 4572 consecutive patients hospitalized for an acute T1MI or T2MI, according to the 3^rd^ universal definition and a prespecified geriatric model of T2MI pathogenesis. In total, 3710 (81%) had T1MI and 862 (19%) T2MI. Patients with T2MI were 10 y older (77 vs 67 y, p<0.001), more frequently female (44 vs 26%, p<0.001) and had more frequent comorbidities. In multivariate analysis, acute heart failure, tachycardia and C-reactive protein elevation at admission were associated with a higher risk of T2MI vs T1MI, whereas chest pain, troponin I peak > 10 µg/L and ST-segment elevation were associated with a lower risk. Underlying mechanisms leading to T2MI highlighted 3 main patterns: 1) Age-related physiological cardiovascular decline 2) chronic predisposing factors including chronic anaemia (10%) and severe aortic stenosis (7%), 3) acute triggering factors, the most common being acute infection (39%), mainly respiratory tract infection, followed by tachyarrhythmia (13%) and acute heart failure (10%). 122 (14%) patients had combined predisposing and triggering conditions for T2MI. In our large population-based survey of T2MI, chronic anaemia and severe aortic stenosis increased predisposition to T2MI and acute respiratory infection was by far the most frequent trigger. Our data shed new light on the age-related pathophysiological basis for discrepancies in oxygen supply and demand leading to MI.

Type 2 myocardial infarction (T2MI), newly redefined by the fourth universal definition of myocardial infarction (MI) [1], is an emerging clinical condition in older patients resulting from a mismatch between supply and demand of myocardial oxygen in the absence of atherothrombosis. This model was initially based on autopsy data which highlighted the lack of thrombi in the coronary arteries of 31% MI deaths [2], and contemporary imaging studies demonstrating the heterogeneity in underlying causes of acute MI [3]. However, pinpointing the clinical conditions leading to T2MI has proven difficult, and distinguishing between myocardial injury, type 1 MI (T1MI) and T2MI remains a major challenge in routine clinical practice [4]. Because coronary angiography is only rarely performed in frail elderly patients, there is a need for clinical markers for every-day distinction between T2MI and T1MI. In contrast with T1MI, corresponding to spontaneous plaque rupture or erosion and resulting in thrombus formation, T2MI pathogenesis is complex and multifactorial: multiple and heterogeneous situations may co-exist and lead to a myocardial supply/demand mismatch. Criteria for defining T1MI have varied markedly form study to study. As a consequence, variations in the prevalence of T2MI ranging from 1.6% to 74% have been reported in the literature [5,6]. Although several specific clinical criteria have been proposed for the practical application of the universal definition [7,8], only few prospective data are available to date to support these criteria [9,10] and investigation of the underlying mechanisms is poor. In particular, the potential combination of acute and chronic clinical conditions promoting the development of oxygen mismatch has never been explored.

From a large multicentre contemporary prospective survey, we aimed to comprehensively describe the factors leading to T2MI and to propose a new model of etiologic classification using a geriatric approach of pathogenesis.

## MATERIALS AND METHODS

### Patients

Characteristics of the French regional *obseRvatoire des Infarctus de Côte d'Or* (RICO) survey have previously been described [11]. Briefly, RICO is an ongoing survey that prospectively collects data from patients hospitalized for acute MI in the cardiology intensive care units of teaching hospitals, general hospitals, and private clinics receiving acute MI emergencies for one region of eastern France. From October 1st, 2012 to March 31st, 2017, all consecutive patients admitted for type 1 or type 2 MI within 24βhours after symptom onset were included in the present study. T1MI or T2MI was prospectively adjudicated according to third universal definition [12]. Patients with myocardial injury, Takotsubo cardiomyopathy, type 3, 4 or 5 MI were excluded from the analysis.

The present study complied with the Declaration of Helsinki and was approved by the Ethics Committee of Dijon University Hospital. Each patient gave written consent before participation.

### Data collection

Demographic data, cardiovascular risk factors and history were collected for all patients as were on-admission ECG, clinical and biological data, as described previously [11]. Length of intensive care unit (ICU) stay and GRACE score for mortality risk [13] were also included [13]. Blood samples were taken on admission to measure haemoglobin and C-reactive protein levels, plasma NT proBNP, Low Density Lipoprotein (LDL) cholesterol and serum creatinine. The estimated glomerular filtration rate was calculated using the Chronic Kidney Disease-EPIdemiology Collaboration formula (CKD-EPI). Cardiac troponin I peak (Dimension Vista LOCI assay) [14] was assessed by sampling every 8 hours during the first 2 days after admission. Left ventricle ejection fraction was measured by echocardiography. Coronary angiography data, including normal and non-obstructive coronary artery (MINOCA) [15] rates and rate of reperfusion (percutaneous coronary intervention, coronary bypass surgery and thrombolysis) were also collected.

**Figure 1.**
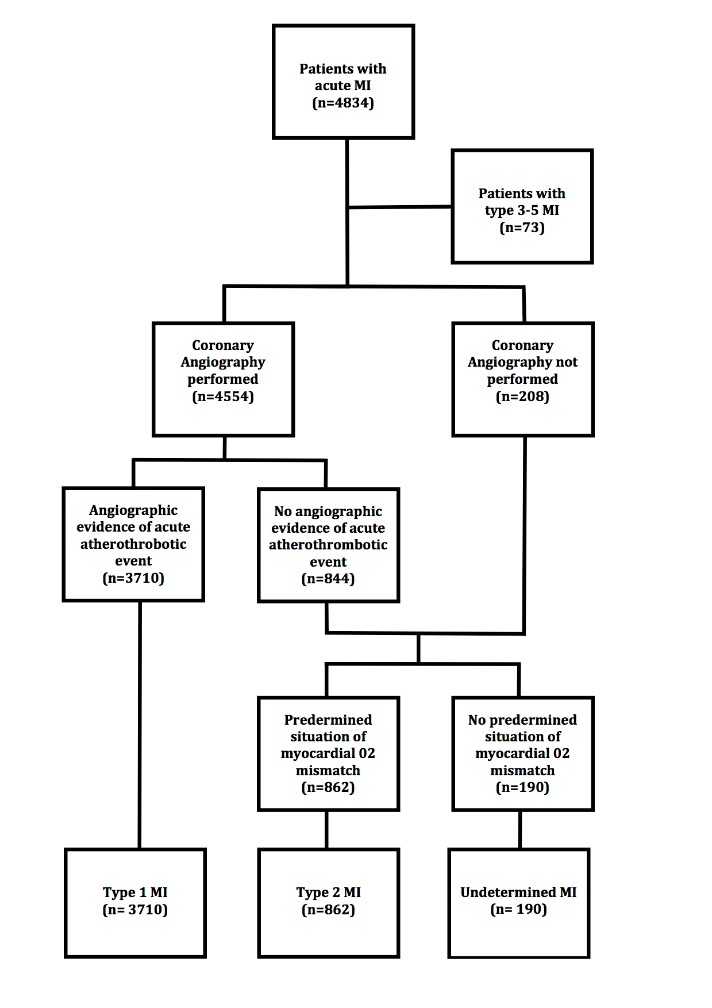
Flow chart.

### Identification of Type 2 MI cases

Each case was reviewed and adjudicated by two independent reviewers (one cardiologist and one internist) with a 85% inter-observer agreement. Any discrepancies were resolved by consensus after an in-depth review of the patient’s medical records. MI were defined according to the third universal definition established in 2012, i.e. rise of cardiac troponin above the 99^th^ percentile upper reference limit and with at least one of the following: symptoms of ischemia, new significant ST changes or new left bundle branch block, development of pathological Q waves [12]. T1MI was defined as MI related to ischemia due to a primary coronary event such as spontaneous plaque erosion or rupture, intraluminal thrombus or coronary dissection. T2MI was defined by the absence of evidence of plaque rupture at coronary angiography when available and at least one of the conditions listed below at the onset of MI symptoms [7,8]. Study flow chart and T1MI/T2MI adjudication criteria are presented in Figure 1. Conditions were identified according to previous publications, covering the whole spectrum of T2MI cases and classified as chronic (predisposing) or acute (precipitating) factors.

*Predisposing factors*

– Severe aortic stenosis [10], diagnosed by doppler echocardiography and defined according to current guidelines [16]
– Hypertensive cardiomyopathy [7], defined as essential hypertension with a systolic blood pressure (BP) >160 mmHg and concomitant left ventricular hypertrophy identified by echocardiography and/or ECG
– Thyrotoxicosis [10], defined as documented clinical symptoms of hyperthyroidism associated with elevated peripheral hormones
– Chronic severe anaemia [7], defined as haemoglobin < 5.5 mmol/L for men and < 5.0 mmol/L for women (measured on admission) and/or the need to use blood products.

*Precipitating factors*

– Active bleeding [8], defined as acute external bleeding with haemoglobin rate < 5.5 mmol/L for men and < 5.0 mmol/L for women and/or the need to use blood products.
– Acute respiratory failure [7], defined as clinical signs lasting ≥ 20 minutes with an arterial oxygen tension < 60 mmHg, from non-cardiogenic and non-infectious causes;
– Severe acute heart failure occurring prior to MI symptoms, including: 1) Acute pulmonary oedema [7], defined as the presence of signs of pulmonary oedema, and need for treatment with nitrates or diuretics, 2) cardiogenic shock [7], defined as systolic BP < 90 mmHg and/or diastolic BP < 60 mmHg associated with evidence of systemic hypo-perfusion (e.g. hyperlactatemia) and low cardiac output.
– Bradyarrythmia [7], requiring medical treatment or cardiac pacing.
– Peri-operative context [17], defined as MI occurring within the first 48h after surgery.
– Coronary spasm [12], refers to a sudden, intense vasoconstriction of an epicardial coronary artery that causes vessel occlusion or near occlusion on coronary angiography, even in the absence of stimulation.
– Coronary embolism [7], defined as a high thrombus burden despite a relatively normal underlying vessel or recurrent coronary thrombus (left heart endocarditis, intracardiac mural thrombus, documented venous thrombus, and a patent foramen ovale or atrial septum defect).
– Acute infection [8], defined as a clinical diagnosis of acute infection by the physician, with at least one of the following: fever > 39°C, tachypnea > 24 breaths/min, tachycardia > 100 beats/min, leukocytes > 12.10^9^/L
– Supraventricular tachyarrhythmia (i.e. atrial fibrillation or flutter) lasting ≥ 20 min with a ventricular rate > 150 beats/min [7].
– Ventricular tachycardia lasting ≥ 20 min [7].
– Convulsive seizure in the presence of generalized tonic- clonic seizure, regardless of its duration [18].

### Model definition

Given the advanced age of patients with T2MI (median age 77 y) and their frequent comorbidities, we aimed to apply a geriatric functional model [19] to T2MI pathogenesis. In this model, functional failure (T2MI is here considered as an acute coronary failure) results from the superimposition of three factors:

1) Cardiovascular aging (mainly progression of coronary atherosclerosis), which *per se* is not responsible for functional failure whatever the age.
2) Predisposing factors related to chronic diseases (i.e. severe chronic anaemia, severe aortic stenosis, thyrotoxicosis, hypertensive cardiomyopathy).
3) Acute potentially preventable triggering factors for decompensation (e.g. acute infection, tachyarrhythmia, acute respiratory failure).

**Figure 2.**
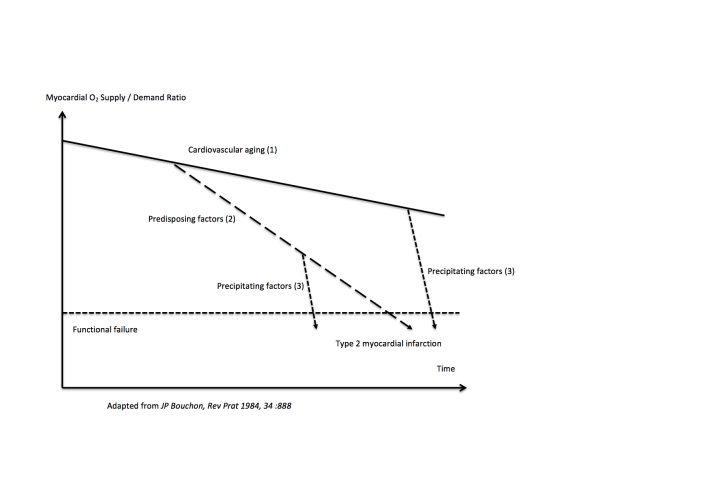
Geriatric model applied to type 2 myocardial infarction pathogenesis.

Both predisposing and precipitating factors, either combined or separate, may be responsible for functional failure. This model is presented in Figure 2.

### Statistical analyses

Continuous variables were expressed as mean ± standard deviation or median and interquartile range. A Kolmogorov-Smirnov test was performed to analyse the normality of continuous variables. Student’s t-test or the Mann-Whitney test was used to compare continuous variables. Chi 2 or the Fisher’s test was used to compare dichotomous data. The threshold for significance was set at 5%. SPSS version 12.0.1 (IBM Inc, USA) was used for all statistical analysis.

## RESULTS

### Baseline characteristics

Among the 4572 patients, 3710 (81%) had T1MI and 862 had T2MI (19%) (Table 1). Overall, T2MI patients were 10 years older (median age 77 [65-84] vs 67 [56-79] y, p<0.001), more frequently female (44% vs 26%, p<0.001) had more comorbidities and cardiovascular history (*i.e*. coronary artery disease: 37% vs 23%, p<0.001) and subsequently more chronic cardiovascular treatments (*i.e.* anticoagulants or antiplatelet medications) than T1MI patients. Acute clinical presentation was more severe in T2MI (i.e. heart failure: 46% vs 22%, p<0.001 and GRACE risk score 171 [138-197] vs 143 [120-170], p<0.001). ECG at admission showed less frequent ST segment elevation (24% vs 52%, p<0.001) and a higher rate of atrial fibrillation or flutter (19% vs 9%, p<0.001). Troponin Ic peak was much lower (3 [1-14] *vs* 17 [3-68] µg/L), although NT-proBNP level was markedly higher (3132 [729-10246] vs 578 [150-2334] pg/mL) in T2MI patients. Troponin peaks remained lower in T2MI group compared with T1MI group for both ST-segment elevation MI (STEMI) (respective median troponin Ic peak: 9.6 vs 49 µg/L, p<0.001) and non STEMI patients (2.7 vs 4.9 µg/L, p<0.001) Median time from symptoms onset to admission was 30 min shorter in T2MI patients (p<0.001). Coronary angiography was more common in cases of T1MI (p<0.001). Among patients with angiography data, MINOCA accounted for 37% of T2MI versus 1% of T1MI. Three-vessel or main left disease was similar for T1MI and T2MI groups. Revascularization procedures were used in 30% of T2MI patients and coronary artery bypass surgery rate was similar for both groups. All-cause and cardiovascular in-hospital deaths were twice as frequent among T2MI patients (11% vs 5% and 9% vs 5%, respectively, p<0.001).

**Figure 3.**
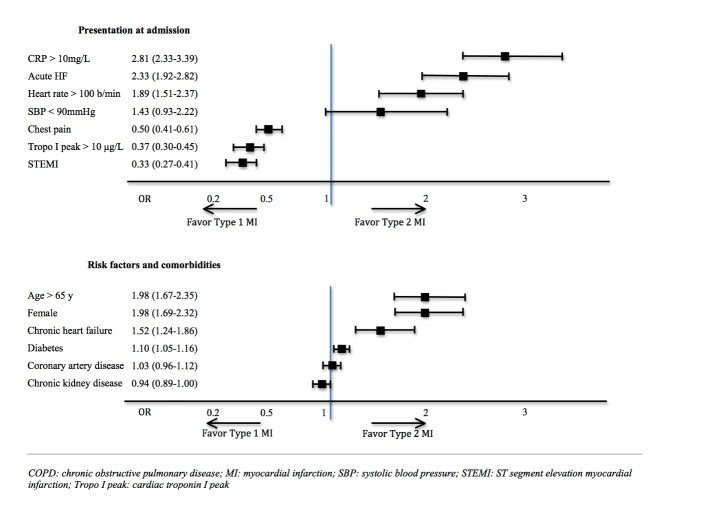
**Multivariate analysis of factors associated with type 2 myocardial infarction**. CRP: C-reactive protein; HF: heart failure; MI: myocardial infarction, SBPL systolic blood pressure; STEMI: ST-segment elevation myocardial infarction; Trpo I peak: cardiac troponin I peak.

Multivariate analysis of factors associated with T2MI versus T1MI is presented in figure 3. Acute heart failure, C-reactive protein elevation and tachycardia were associated with a higher risk of T2MI whereas chest pain, high Troponin I peak and ST-segment elevation were associated with a higher risk of T1MI. Older patients, women, and patients with chronic heart failure or diabetes were groups at higher risk of T2MI; patients with chronic kidney disease tend to have a higher risk of T1MI.

### T2MI Etiologic analysis

**Figure 4.**
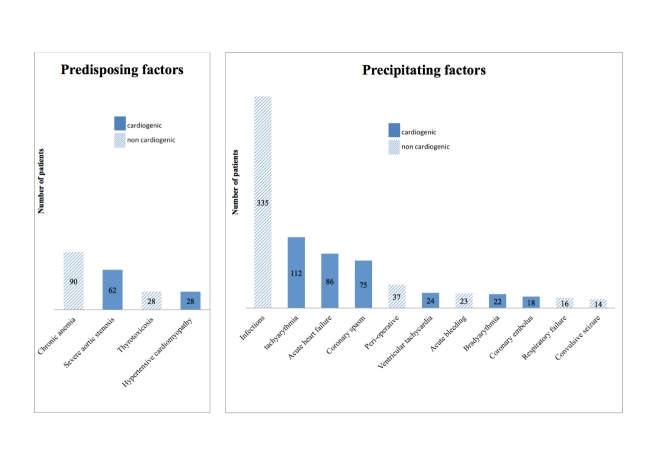
Predisposing and precipitating factors for type 2 myocardial infarction.

Among the 862 cases of T2MI, 208 predisposing and 764 precipitating situations were identified with the prospective adjudication procedure (Fig. 4):

The main predisposing factors were chronic severe anaemia (10%) and severe aortic stenosis (7%).

Acute infection was by far the most common acute precipitating factor (39%), and mainly included respiratory infection (26%), followed by supraventricular tachyarrhythmia (13%) and acute heart failure (10%).

Main characteristics of T2MI patients with predisposing thyrotoxicosis or presenting an acute infection, acute heart failure, or convulsive seizures as T2MI trigger are presented in table 2.

**Figure 5.**
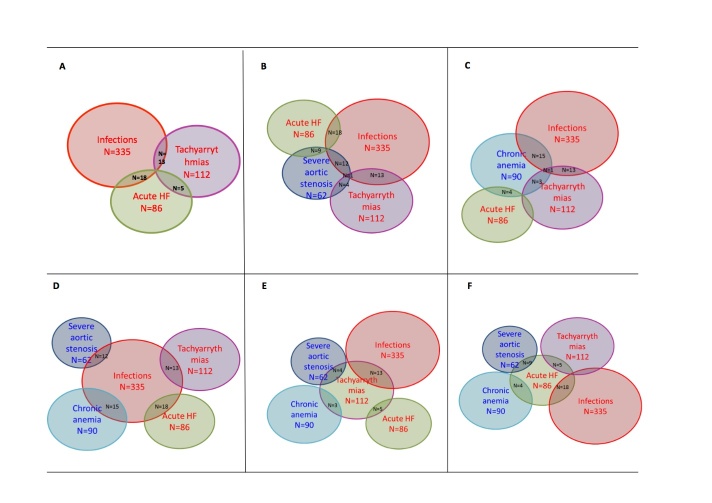
**Main combinations of etiologic factors for type 2 acute myocardial infarction**. **(A)** Combination of acute factors. (B and C) Combinations of acute and chronic factors. **(D)** Acute and chronic factors combined with infection. **(E)** Acute and chronic factors combined with tachyarrhythmia. F. Acute and chronic factors combined with acute heart failure.

Interestingly, 122 (14%) patients had combined chronic and acute conditions for T2MI. Among predisposing factors, chronic severe anaemia and severe aortic stenosis were the most frequent factors combined with acute triggers, which were mainly infection and acute heart failure. The main associations between etiological factors are shown in Figure 5.

## DISCUSSION

The present study is to date one of the largest real-life studies to comprehensively analyse the whole spectrum of clinical features for T2MI patients, using a comprehensive geriatric approach of pathogenesis. To our knowledge, only Saaby *et al*. have clearly proposed a set of etiological criteria and cut-offs for T2MI adjudication, reflecting an imbalance between the supply and demand of myocardial oxygen [7]. However, our large database allows a wider evaluation of less frequent clinical situations, as initial restrictive criteria could have led to an under-evaluation of T2MI frequency. T2MI was identified in 7.3% of patients with elevated troponins in an emergency setting [9], which is markedly lower than other studies for which T2MI adjudication was at the experts’ discretion [20]. A more comprehensive and reproducible approach is needed to simplify the diagnosis procedure for clinicians and to harmonise research. Using the French geriatric model of Bouchon [19], we propose here a new approach for T2MI pathogenesis, considering T2MI as a functional cardiovascular failure, resulting from age, combining chronic predisposing and acute conditions for decompensation. Data from a large validated MI survey and a systematic adjudication procedure were used to test this model. Moreover, our study extends T2MI pathogenesis to additional factors, which may contribute to myocardial supply/demand mismatch.

The main results are as follows:

– Chronic anaemia and severe aortic stenosis are chronic situations at risk for T2MI, in particular when associated with acute infections or tachyarrhythmia.
– The leading acute decompensating factor for T2MI was non-cardiogenic, *i.e.* infection and particularly respiratory tract infection,
– Thyrotoxicosis, regardless of tachyarrhythmia, emerged as a major underlying mechanism, exhibiting the same prevalence as hypertensive cardiomyopathy.

Here, the characteristics of T1MI and T2MI and their relative proportions are consistent with previous studies. T2MI patients were 10 year older, more often women, had more cardiovascular risk and comorbidities than patients with T1MI [8,9,18]. Moreover, troponin Ic peak was lower [20], and ST segment elevation was much less frequent (24%) [10]. In contrast, rhythm and conduction disorders, including atrial fibrillation and left bundle branch block were more frequent [21]. Patients with T2MI had higher GRACE scores than T1MI patients, and in-hospital mortality was twice as high [10]. Surprisingly, time from symptoms onset to admission was shorter for T2MI patients, despite the less frequency of ST segment elevation. One explanation could be the more severe clinical presentation, especially the more frequent acute heart failure, resulting in a faster alerting time and a prompter medical support.

Our findings are also consistent with retrospective studies for some of the leading causes of T2MI (i.e. tachy-/brady-arrhythmias, severe aortic stenosis, hypertensive cardiomyopathy, cardiogenic shock, severe respiratory failure, severe anaemia, coronary spasm, and coronary embolism) which have been widely described, and are included as part of the initial definition [12] and confirmed in the new definition [1]. Notably, only patients with definite MI criteria (i.e. troponin elevation with chest pain and/or ECG changes) were considered in this study even though T2MI causes mentioned above are also frequently responsible for myocardial injury without infarction (i.e. troponin elevation without such signs), named non-ischemic myocardial injury (NIMI) in the new universal definition of MI [1]. The distinction between T2MI and NIMI is often difficult in clinical practice but appears to be of low prognostic value [20]. To date, the therapeutic strategy of T2MI and NIMI has still to be determinated [1].

Anaemia, a widespread condition in the elderly, was one of the leading causes of T2MI in the literature [9,10,21]. Our findings reveal that severe anaemia was the main predisposing factor (10%) and was often combined with other contributing factors. Therefore, patients with severe anaemia are at increased risk for T2MI, in particular if there is concomitant infection (17%) or, more rarely, acute heart failure or tachyarrhythmia. However, acute bleeding and chronic anaemia should be categorized separately, as patient characteristics, myocardial tolerance and prognosis largely differ [22]. In older anaemic patients, an association between red blood cell transfusion in the acute phase of MI and decreased mortality was recently highlighted [23], suggesting the potential benefit of transfusion following anaemia in T2MI.

Acute heart failure was the second acute cardiogenic precipitating factor, often associated with infections (21%) and chronic conditions such as anemia or severe aortic stenosis.

Surprisingly, even though it is the most frequent cause of T2MI in a number of studies [5] including our own, infection was not reported as an etiologic factor in the 4^th^ Universal Definition of MI [1]. Notably, we observed that infection was the acute precipitating factor the most often associated with other contributing factors, *i.e.* either chronic (anaemia and severe aortic stenosis) or acute (acute HF or tachyarrhythmia) conditions. Moreover, our data show that infections of the respiratory tract are the infection type at higher risk for T2MI. However, we found that other types of sepsis, including urinary tract infections, are also potential triggering mechanisms for T2MI, and this hypothesis has also been put forward for T1MI [24]. Infection could prompt both a decrease in O_2_ supply via hypoxemia and an increase in myocardial consumption of O_2_ because of sepsis-related high cardiac output [25]. This could explain in part the increased hibernal incidence of MI [24] and the protective effect of influenza [26] and pneumococcal [27] vaccinations.

We have observed additional causes that were less known and have never been specifically addressed in such large series of cases. Thyrotoxicosis has only been previously reported in 4 patients by Stein *et al.* [10]*.* Our work shows that overt hyperthyroidism, although very rare in the community-dwelling older population [28], is the second leading non-cardiogenic predisposing factor, behind anaemia. Myocardial ischemia has already been linked to an increase in myocardial oxygen demand in response to the increase in myocardial contractility and workload associated with elevated thyroid hormone levels (i.e. elevated T4, T3, or both) [29].

Hyperthyroidism has also been shown to cause severe, diffuse left-sided coronary vasospasm resulting in MI and/or fatal arrhythmia, especially in young females, even in the absence of coronary artery disease [30]. Interestingly, successful treatment of hyperthyroidism has been associated with a reversal of these symptoms [29].

Convulsive seizure, a rare acute condition (2%) preceding T2MI in the present study, has been scarcely described, as case reports, in both T1MI [31] and T2MI [18,31]. A massive catecholamine release, similar to what is observed in Takotsubo cardiomyopathy induced by ischemic stroke or epileptic events [32], could explain an increased myocardial oxygen demand, as shown by the concomitant tachycardia [31].

Peri-operative T2MI has only rarely been described. Notably, a retrospective review reported that non-cardiac surgery was the most common associated setting for T2MI or myocardial injury (38%) [33]. In contrast, our prospective study reports a lower prevalence -at 5%- of precipitating conditions among patients hospitalized in cardiology ICU. While T1MI in the post-operative setting is rare, myocardial oxygen supply/demand balance can be altered [17]. Post-operative T2MI most likely has multiple pathophysiological pathways, including tachycardia, acute bleeding, and hypoxemia.

Severe aortic stenosis has been associated with T2MI [2,10,33]. In the present study, this chronic predisposing factor was the second most frequent predisposing factor of T2MI, and was often associated with acute conditions (infections, acute heart failure or tachyarrhythmia). Experimental works in patients with severe aortic stenosis undergoing aortic balloon valvuloplasty have shown that hemodynamic stress decreases aortic pressure (thus decreasing coronary perfusion and oxygen supply) and increases left ventricular pressure (increasing oxygen demand) [34]. Further studies are needed to fully understand the pathophysiological link between aortic stenosis and acute situations at risk of MI triggering.

### Study limitations

The present study has several limitations. First, this work was limited to patients hospitalized in cardiology ICU, and is thus difficult to extrapolate to other hospital departments where T2MI incidence and characteristics may be different [7]. Secondly, even if coronary angiography was available for a large proportion of patients, this examination has a weak sensibility for small or eccentric thrombus. Coronary plaque rupture and ulceration could thus have been under-diagnosed, and no other imaging techniques, such as intravascular ultrasound, were used, resulting in potential misclassifications of T2MI [5]. Third, some of the criteria for T2MI adjudication are debatable, including severe anaemia. Because anaemia is a widespread condition in older adults, we deliberately choose a restrictive haemoglobin cut-off in order to avoid overestimation chronic anaemia burden, which has been done in past studies as well [7]. Fourth, since the beginning of the study, a change in MI definition has occurred [1,12]. Notably, myocardial injury has been clearly distinguished from MI by the fourth universal definition. However, only patients with clinical, imaging or ECG signs of myocardial ischemia are included in the RICO survey, which corresponds to the new definition of MI, and non-ischemic myocardial injury have thus not been included in this study. Fifth, several criteria of T2MI, including acute infections, have not been acknowledged as pathophysiological factors by the 3rd and 4th universal definitions. Consistently with previous studies, we believe that those criteria are however responsible for an ischaemic imbalance of oxygen, defined as the pathophysiological pattern of T2MI by 4^th^ the universal definition. Finally, despite the rigorous use of systematic diagnostic criteria for each case by two expert investigators, the clinical situations leading to T2MI are probably too polymorphic and multifactorial to be reduced to specific criteria. Because our model does not aim to be exhaustive, the frequency of T2MI has probably been underestimated.

### Conclusions

T2MI stems from a number of heterogeneous underlying factors, including age-related cardiovascular aging. In our large population-based study, we used a prospective, comprehensive and analytic strategy to characterize patients with T2MI hospitalized in cardiology ICU. We identified older age, acute heart failure, tachycardia and C-reactive protein elevation as associated with T2MI, whereas chest pain, STEMI and high troponin elevation were more frequent in T1MI. Our findings have led to propose a new model for T2MI, based on a geriatric-derived pathogenesis and including chronic and acute conditions, which can be superposed in some cases. We showed that respiratory infections are by far the most common acute decompensating factor, as they were found in more than a quarter of T2MI patients. Among predisposing factors, severe anaemia and aortic stenosis are the leading contributors. The characterization of contributing factors and their mutual interactions is a key step for understanding myocardial oxygen imbalance and improving the management of T2MI, frequently encountered in older frail patients. Our findings may lead to the development of personalized treatment strategies and better outcomes for T2MI patients.
